# A Case of Cushing's Syndrome due to Ectopic Adrenocorticotropic Hormone Secretion from Esthesioneuroblastoma with Long Term Follow-Up after Resection

**DOI:** 10.1155/2018/6389374

**Published:** 2018-02-04

**Authors:** Leslee N. Matheny, Sudipa Sarkar, Hanyuan Shi, Jiun-Ruey Hu, Hannah Harmsen, Ty W. Abel, Shubhada M. Jagasia, Shichun Bao

**Affiliations:** ^1^Vanderbilt University Medical Center, Division of Endocrinology, Department of Medicine, Vanderbilt University, 1215 21st Avenue South, Nashville, TN 37232, USA; ^2^Johns Hopkins University School of Medicine, Division of Endocrinology, Diabetes and Metabolism, 5501 Hopkins Bayview Circle, Baltimore, MD 21224, USA; ^3^Vanderbilt University Medical Center, Department of Surgery, Vanderbilt University, 1161 21st Avenue South, Nashville, TN 37232, USA; ^4^Vanderbilt University School of Medicine, 2215 Garland Ave, Nashville, TN 37232, USA; ^5^Vanderbilt University Medical Center, Department of Pathology, Microbiology and Immunology, 1161 21st Avenue South, Nashville, TN 37232, USA

## Abstract

We present a case of a 52-year-old male who developed Cushing's Syndrome due to ectopic adrenocorticotrophic hormone (ACTH) secretion from a large esthesioneuroblastoma (ENB) of the nasal sinuses. The patient initially presented with polyuria, polydipsia, weakness, and confusion. Computed tomography scan of the head and magnetic resonance imaging showed a 7 cm skull base mass centered in the right cribriform plate without sella involvement. Work-up revealed ACTH-dependent hypercortisolemia, which did not suppress appropriately after high-dose dexamethasone. Subsequent imaging of the chest, abdomen, and pelvis did not reveal other possible ectopic sources of ACTH secretion besides the ENB. His hospital course was complicated by severe hypokalemia and hyperglycemia before successful surgical resection of the tumor, the biopsy of which showed ENB. Postoperatively, his ACTH level dropped below the limit of detection. In the ensuing 4 months, he underwent adjuvant chemoradiation with carboplatin and docetaxel with good response and resolution of hypokalemia and hyperglycemia, with no sign of recurrence as of 30 months postoperatively. His endogenous cortisol production is rising but has not completely recovered.

## 1. Introduction

Esthesioneuroblastoma (ENB), or olfactory neuroblastoma, is a tumor of the nasal and paranasal sinuses, derived from the olfactory neuroepithelium [1, 2] and represents only 3–6% of all cancers in the nasal cavity and paranasal sinuses [2, 3]. Paraneoplastic ENB is rare and only has been reported in a handful of cases in the literature. One type of paraneoplastic ENB is ectopic adrenocorticotrophic hormone (ACTH) secretion. Ectopic ACTH secretion in ENB is highly unusual and can lead to severe symptoms of Cushing's Syndrome (CS) including persistent hypertension, hypokalemia, hyperglycemia, and opportunistic infections. It is important to be cognizant and recognize the pathophysiology, work-up, and treatment of ACTH-secreting ENB in its varied presentations. We present a case here for that discussion.

## 2. Case Presentation

**Figure 1:**
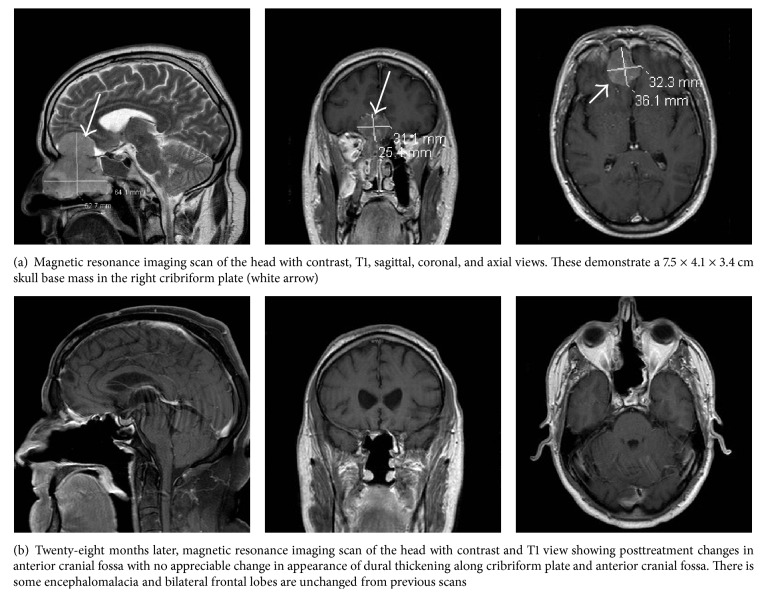


A 52-year-old Caucasian male with a past medical history of hypertension presented to our hospital for planned resection of a large skull base mass of the right cribriform plate by the neurosurgery service. He had initially presented at an outside hospital with anosmia and right nasal airway obstruction. He was diagnosed with new onset diabetes mellitus. Computed tomography (CT) scan of the head and magnetic resonance imaging (MRI) of the face revealed a 7.5 × 4.1 × 3.4 cm mass of the right cribriform plate, extending intracranially into the right anterior cranial fossa and displacing the frontal lobe with no sellar involvement. Imaging at our center confirmed the findings (Figure 1(a)). However, on the planned day of procedure, his labs were significant for severe hypokalemia with a potassium of 2.0 mmol/L (normal range: 3.3–4.8) and metabolic alkalosis with arterial pH of 7.64 (7.35–7.45) and serum HCO_3_^−^ of 44 mmol/L (21–29). Surgery was postponed, and the endocrinology service was consulted. It was noted that the patient had been experiencing several weeks of severe weakness, polyuria, and more than 20 pounds of weight loss before the scheduled operation. He was initially treated with oral and intravenous potassium chloride (KCl), but his serum potassium continued to be refractory to acute repletion. In addition, he had increased insulin requirement to control his serum glucose levels.

As part of his hypokalemia work-up, he was found to have a significantly elevated random plasma cortisol of 1851 nmol/L and plasma ACTH of 152 pmol/L (1.5–11.2). 24-hour urine free cortisol was also grossly elevated at 32,027 nmol/day (<165). His renin and aldosterone levels were normal. His TSH was also normal. Thus, CS was suspected, and imaging was ordered to locate possible sources. CT scan of his chest, abdomen, and pelvis noted bilateral adrenal enlargement, but no distinct nodules and no apparent sources of ACTH secretion were found. This raised the possibility of ENB being the ectopic source. High-dose (8 mg) dexamethasone suppression testing was performed, with the next-day morning cortisol of 1895 nmol/L, which was not suppressed at all, and ACTH was still elevated at 75.7 pmol/L. He continued to be aggressively treated with KCl. Eplerenone, magnesium oxide, and ketoconazole were also added. On day 5, his potassium was stabilized in the low-normal range. He was discharged on day 6, with a plan for a definitive resection of the tumor in 3 weeks, after the Christmas and New Year holidays.

**Figure 2:**
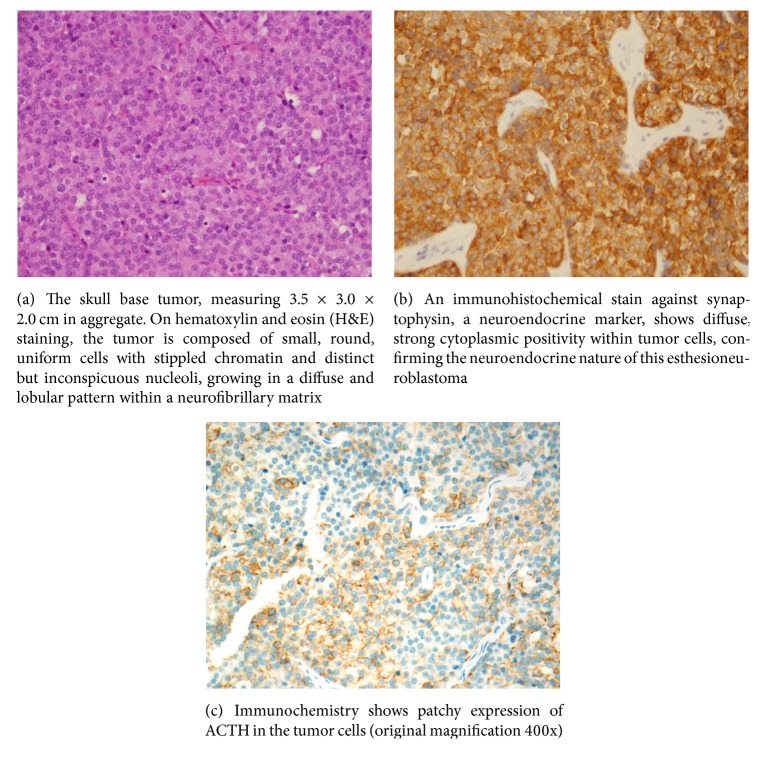
Histology staining of tumor.

However, the patient was readmitted 3 days later, for confusion, hypotension, and continued hypokalemia refractory to his oral potassium medications. He was once again aggressively potassium repleted, and surgery was performed a week later. A bifrontal craniotomy was performed by the neurosurgery team and bilateral maxillary antrostomy, ethmoidectomy, and sphenoidotomy were performed by otolaryngology along with resection of the tumor under endoscopic guidance. The mass was revealed to be a mixed-consistency lesion eroding through the anterior right planum extending through the dura. Intraoperative biopsy showed that the tumor contained lobular growth of small round cells with uniform hyperchromatic chromatin, inconspicuous nucleoli, and scant fibrillary cytoplasm, with rare mitotic figures present (Figure 2(a)). The cells stained positive for chromogranin, synaptophysin, and CD56 on histology, with S100 highlighting the periphery of the clusters (Figure 2(b)). Final confirmation was done with ACTH-staining, which was positive in the tumor cells (Figure 2(c)). The superior septum and anterior inferior septum were involved by tumor, but the tumor was not present on deeper permanent sections.

**Figure 3:**
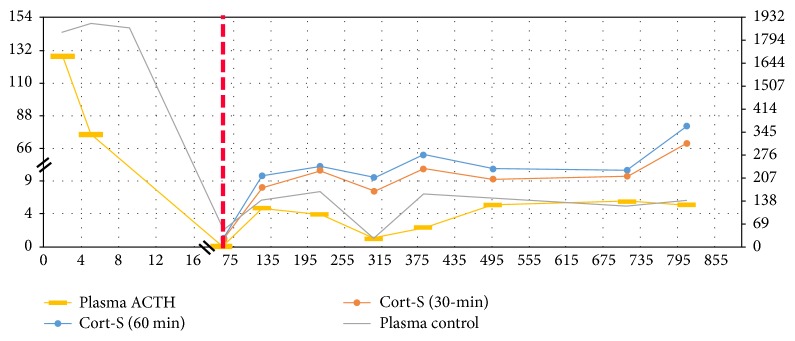
Cortisol and ACTH changes before and after operation. The *x*-axis details the day since initial admission to our hospital. Day 19 or the red line is the resection of the esthesioneuroblastoma. The left *y*-axis is the level of plasma adrenocorticotropic hormone (ACTH (pmol/L)); the right *y*-axis is the level of plasma cortisol (Cort-S (nmol/L)). At his outpatient follow-up, 0.25 mg ACTH stimulation tests were done in clinic with assessment of endogenous cortisol production (plasma cortisol level measured at baseline and 30 and 60 minutes after ACTH stimulation).

The postoperative course was uncomplicated, with decreasing daily potassium and insulin requirements and resolution of metabolic alkalosis (Table 1). He no longer required ketoconazole or eplerenone but needed glucocorticoid for central adrenal insufficiency as ACTH dropped to undetectable level at < 1 pmol/L the day after surgery (Table 2). Shortly after, the patient underwent adjuvant chemotherapy with carboplatin and docetaxel as well as intensity-modulated radiation therapy for 7 weeks. He was treated on maintenance doses of dexamethasone 0.5 mg daily and then 0.25 mg daily and eventually tapered down to hydrocortisone 15 mg daily for his adrenal insufficiency. He was tapered off insulin 3 months after surgery, with good diabetes control on metformin 500 mg twice daily alone. He was determined to be in complete remission as of his most recent visit to medical oncology, and MRI scans of the brain and face have been negative for recurrence 28 months after the initial resection (Figure 1(b)). Although his hypokalemia has resolved, his endogenous cortisol production is improving but has not yet recovered completely (Figure 3). ACTH stimulation tests revealed that stimulated cortisol at 60 minutes was 52.4 nmol/L at 3 months, 209.7 nmol/L at 9 months, 234.5 nmol/L at 12 months, 231.7 nmol/L at 16 months, and 364.1 nmol/L at 26 months, with normal cut-off being greater than 500 nmol/L.

## 3. Discussion

This patient was diagnosed with ectopic ACTH syndrome (EAS) based on the preoperative findings of high plasma ACTH concentration, random serum cortisol, and 24-hour urinary cortisol. Other ectopic sources were ruled out by CT scan. At this point, it was strongly suggested that the ENB (Kadish stage B involving two paranasal sinuses) was the source of the ectopic ACTH [4]. Based on these findings and his deteriorating condition, he was emergently taken for resection of the ENB. Pathology of the tumor confirmed synaptophysin positivity on stain, which indicated this was indeed a neuroendocrine neoplasm [5]. This was further confirmed by positive ACTH-staining. Along with the clinical, histological findings and the subsequent improvement in his condition after resection, we confirmed the diagnosis of ectopic ACTH-secreting esthesioneuroblastoma. During his postoperative course, his potassium requirements significantly decreased and he no longer required medication for potassium repletion. Thirty months after resection, the patient still suffers from secondary adrenal insufficiency, but his endogenous cortisol production is improving. He has had no tumor recurrence based on the last surveillance.

Our report demonstrates an unusual presentation of CS due to ectopic ACTH secretion from ENB and adds key data to the literature concerning this rare condition. There are fewer than 25 cases in the literature. We describe an EAS-ENB with comprehensive follow-up up to 30 months after resection, which is one of the longest follow-up periods among studies to date [6, 7]. Our extensive reporting of the initial hospital course and measurements of hypocortisolism after resection has not previously been described.

Our patient required multiple medication dosage adjustments in order to manage his hypokalemia, including oral and intravenous KCl and magnesium, as well as antimineralocorticoid such as eplerenone and steroidogenesis inhibitors such as ketoconazole. These were titrated to give him low-normal potassium levels and to relieve symptoms of muscle weakness and numbness. Other case reports have described usage of metyrapone, etomidate, mitotane, and mifepristone as steroidogenesis inhibitors [8]. As described in our report, this was not enough and he was readmitted to the hospital with severe weakness from electrolyte derangements and altered mental status, where definitive treatment per surgery was emergently performed. In addition, we also saw a significant increase in the patient's insulin requirements that eventually decreased to preadmission units after tumor resection.

The patient was placed on different corticosteroid (dexamethasone and later hydrocortisone) for central adrenal insufficiency the months after tumor resection. Baseline ACTH and cortisol levels remained to be low-normal in our patient. He failed all his ACTH stimulation tests so far (cortisol level <500 nmol/L 60 min after 0.25 mg ACTH stimulation) but has had improvement of stimulated cortisol. The patient does not have clinical symptoms of adrenal insufficiency such as weight loss, cardiovascular collapse, and hypoglycemia. The chronic massive production of ectopic ACTH from EAS has suppressed his endogenous long term cortisol production (over 30 months). Central adrenal insufficiency usually only requires glucocorticoid replacement, not mineralocorticoid, with hydrocortisone preferred as the most physiological option [9].

In conclusion, we report a case of olfactory neuroblastoma with ectopic ACTH secretion that was treated with resection and adjuvant chemoradiation. Given the paucity of this diagnosis, little is known about how best to treat these patients and how best to screen for complications such as adrenal insufficiency and follow-up. Our case adds more data for better understanding of this disease.

## Figures and Tables

**Table 1:** Hospital course.

Laboratory	Day	1	2	3	4	5	6^*∗*^	9^*∗∗*^	10–17	18^§^	19–26^§§^
K+ (mmol/L)		2.0	1.9	2.2	2.8	3.4	3.6	2.3	2.5–3.7	*3.2*	3.1
HCO3− (mmol/L)		44	38	39	32	29	35	35	21–29	*31*	27
pH		7.64								*7.56*	7.55

*Treatments*											
KCl (mEq)		100	200	260	280	200	80	120	(258)	*810*	(135)
Insulin (Units)		68	111	124	131	153	148	138	(115)	*180*	(54)
Eplerenone (mg)			25	75	125	150	50				
Ketoconazole (mg)						200	200		(350)		
Dexamethasone (mg)										*8*	(0.9)

^*∗*^Patient discharged on day 6 with eplerenone 25 mg twice a day, ketoconazole 200 mg twice a day, KCl 40 mEq four times a day, Lantus 50 units daily, and Lispro 28 units three times a day with meals plus sliding scale. ^*∗∗*^Patient readmitted. ^§^Surgical operation (in italic font). ^§§^Patient discharged on day 26, postoperative day 8 on dexamethasone 0.5 mg daily, Lantus 20 units daily, and Lispro 10 units three times a day with meals plus sliding scale. The numbers in parentheses represent the average amount per day over time interval.

**Table 2:** Hormonal changes.

Hormone	Day	2	5	9	19^*∗*^–26	57	120	214	302	382	495	713	810
Cort-S (60 min)						30	215	243	210	276	235	232	364
Cort-S (30 min)						22	179	229	168	235	204	212	312
Random cortisol		1846	1895	1868		52	141	168	25	160	149	124	141
Plasma ACTH (0–13 pmol/L)		128	76		<1	<1	5	4	1	3	6	6	6

Treatments (per day)													
Hydrocortisone Dexamethasone					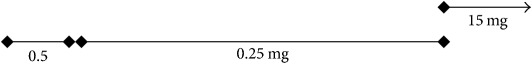								

^*∗*^Day 19 was postoperative day 1; patient was discharged on day 26. Cort-S = cortisol levels during ACTH stimulation test. Normal stimulated cortisol levels should be >500 nmol/L when measured 60 min after intravenous administration of 0.25 mg ACTH.
